# Measurement and analysis of fish pond water temperature field in summer based on fiber Grating string

**DOI:** 10.1371/journal.pone.0317523

**Published:** 2025-01-31

**Authors:** Guoli Li, Fei Feng, Caihua Qian, Bo Wei

**Affiliations:** 1 School of Mechanical and Electrical Engineering, Jinling Institute of Technology, Nanjing, China; 2 School of Mechanical Engineering, Yancheng Institute of Technology, Yancheng, China; 3 Wuxi Brillouin Electronic Technology Co., Ltd., Wuxi, China; University of Kalyani, INDIA

## Abstract

To solve the problems of low detection efficiency and inability to adapt to distributed measurement in traditional detection methods, a water temperature field detection system based on a fiber Bragg grating string was designed. In this system, six fiber Bragg gratings with different center wavelengths are connected in series on a single fiber optic cable based on wavelength division multiplexing technology. The fiber Bragg grating string is encapsulated in stainless steel tube and vertically fixed in the measured water body of the fish pond. The space division multiplexing technology is employed to collect information from multiple fiber Bragg grating strings. Water temperature measurement experiments were conducted in the summer pond environment. The experimental results show that the daily variation curve of temperature in each water layer of the fish pond is relatively smooth and approximates a cosine function with a 24-hour period. In summer, the daily average water temperature in the pond is no more than 1°C higher than the average air temperature. The difference between the maximum and the minimum water temperature is approximately 2°C. During the daytime, the temperature gradually decreases from the surface to the deeper water layers, whereas at night, the temperature variation among the water layers is minimal. As depth increases, the amplitude of the water temperature curve oscillations gradually decreases, exhibiting exponential decay. However, the peak time gradually lags behind. There is a correlation between the temperatures of the water layers in the fish pond, and the smaller the distance between the water layers, the stronger the correlation. The experimental results obtained in this study are highly significant for real-time services in aquatic planting and aquaculture. Additionally, this measurement method can provide valuable reference and guidance for measuring temperature fields in other fluids.

## 1 Introduction

Fish pond water temperature is one of the most important environmental factors for aquaculture and farming. On one hand, water temperature directly affects the reproduction, growth, and distribution of aquatic organisms [1,2]. On the other hand, water temperature indirectly influences the growth of aquatic life through its impact on environmental factors such as dissolved oxygen, pH levels, and ammonia nitrogen content in the water. Water temperature is essential for predicting and understanding pond ecosystem changes [3,4]. It also serves as a critical basis for assessing the normal growth of aquatic organisms. Therefore, detecting pond water temperature fields and studying their variation patterns are important for real-time services in aquaculture and breeding.

Pond water temperature field detection primarily includes two aspects: surface water temperature field detection and vertical temperature field detection. Currently, there are three main types of commonly used methods for water temperature field detection:

1. The contact-based measurement method using electrical sensors and fiber optic sensors. In this method, sensors are usually placed on the water surface or embedded in different depths of water for real-time temperature measurement. The electric quantity sensor used for water temperature measurement has gone through the development stages of sensor technology such as thermistors, platinum resistors, digital integrated temperature sensors, and quartz temperature sensors. Liu et al. [5] utilized multi-sensor information fusion technology to fuse the water temperature results collected by multiple Pt100 thermal resistors and obtained more accurate temperature information. Zeng et al. [6] used Pt100 resistance thermometers to measure water temperature and employed two-dimensional fuzzy control measurement to achieve both measurement and control of water temperature. DS18B20 is a digital integrated temperature sensor, operating as a single-bus device. It supports multi-point networking, allowing for multi-point temperature measurement. In recent years, it has found extensive applications in temperature detection fields such as water temperature [7–10]. The Institute of Oceanography of the Russian Academy of Sciences had developed a drifter equipped with a quartz temperature measurement module, enabling accurate measurement of sea surface temperature [11]. The working principle of a distributed temperature sensor based on optical time domain reflectometry and the backward Raman scattering temperature effect. Embedding the sensing optical fiber into a water body enables the distributed measurement of water temperature [12–14]. In recent years, the rise of smart aquaculture technologies, particularly the application of the Internet of Things, has provided unprecedented solutions for water body monitoring and management. By deploying a network of smart temperature sensors, the system can continuously monitor changes in water temperature and transmit data in real-time to a central system, thereby achieving comprehensive remote monitoring of water environmental parameters [15,16].
2. The noncontact measurement methods based on infrared radiation, ultrasonic waves, and similar techniques. This method is mainly used for measuring the temperature of the water surface or shallow depths. Wang et al. [17] designed an infrared temperature measurement module and conducted experiments on surface water temperature measurement by integrating internet of things technology. The experimental results demonstrated that the infrared temperature measurement system and the buoy-based temperature measuring instrument exhibited consistent overall trends in their measurements, with a correlation coefficient of 0.98. Hideyuki [18] installed uncooled thermal infrared sensors on unmanned aerial vehicles and utilized thermal infrared imaging technology to achieve measurement and prediction of river water surface temperature. Tohmyoh et al. [19] utilized ultrasonic reflection technology to obtain shallow water temperature and confirmed a strong correlation between the temperatures measured using this technique and those measured using thermocouples.
3. The simulation measurement method based on water temperature field prediction models. This method involves analyzing the relationship between water temperature field and air temperature, and combining observed and recorded historical water temperature data to establish a prediction model for simulating and measuring the water temperature field. Luo Wei et al. [20] employed stepwise regression analysis based on water temperature and concurrent air temperature data to construct predictive models for average and maximum temperatures at different water layers of fish ponds. The model validation results demonstrated an average absolute error in predictions ranging from 0.3 to 0.8°C. Sun et al. [21] constructed a lake and reservoir water temperature forecasting model based on the ensemble Kalman filter algorithm and the CE-QUAL-W2 model. This model exhibits high accuracy in medium to short-term water temperature forecasting. Hao et al. [22] developed a novel deep learning model to extract nonlinear dynamics from climate variables for achieving more accurate predictions of lake surface water temperature.

Resistance thermometers, digital integrated temperature sensors, quartz temperature sensors, and other electrical sensors have drawbacks such as being susceptible to electromagnetic radiation and corrosion. Additionally, their poor stability in long-distance signal transmission makes them unsuitable for distributed measurements. Non-contact measurement methods based on infrared radiation, ultrasonic waves, and similar approaches are only suitable for surface or shallow temperature measurements in water bodies. Moreover, the data processing involved in these methods is complex, and they suffer from poor real-time measurement capabilities. Measurement methods based on predictive models are suitable for water temperature field prediction but cannot achieve real-time temperature measurements.

Compared with electrical sensors, distributed fiber optic temperature sensors based on the backward Raman scattering temperature effect have several advantages, including small size, easy reusability, resistance to electromagnetic interference, and corrosion resistance. However, they have lower spatial resolution and positioning accuracy.

Compared with other equipment in water temperature measurement studies, Fiber Bragg grating (FBG) temperature sensor has the advantages of small size, corrosion resistance, electromagnetic interference resistance, good real-time performance, and high positioning accuracy. Multiple gratings of different wavelengths connected in series on a single optical fiber can achieve distributed measurement. In recent years, researchers have conducted extensive studies on using FBG strings for distributed temperature measurements [23–27].

The fluid temperature field is complex and variable, making it difficult for traditional temperature detection methods to meet the requirements for real-time and distributed measurements. To solve the above problems, a water temperature field measurement method based on FBG string and space division multiplexing technology was proposed. Experiments were conducted on fish pond water during the summer, and the resulting data were analyzed. In this method, FBG strings are arranged at different depths of the water body in a fish pond for real-time temperature measurement in multiple water layers. The distribution patterns and changing patterns of water temperature fields in the fish pond, and their relationship with the solar radiation power and other factors were studied. This method enhances the precision and efficiency of water temperature field measurements and also serves as a reference and inspiration for temperature field measurements in other fields.

## 2 Theoretical analysis of water temperature field

The temperature of a body of water depends on the thermal exchange process between the water and its surrounding medium. The sources of heat gained by a body of water include solar radiation, atmospheric radiation, inflow heat, and internal heat within the water. Among these, the heat generated internally within the body of water is relatively small. Heat loss from a body of water includes the heat carried away by outflow, the longwave radiation emitted by the water, and the heat loss caused by water surface evaporation. Additionally, the direction of heat conduction between the water body and the atmosphere, as well as the heat exchange between the water body and the adjacent soil, is variable. There are many factors that influence the temperature distribution within a body of water, including its flow dynamics, water transparency, and geometric shape.

Compared to large bodies of water such as oceans, rivers, lakes, and reservoirs, the characteristics of fish ponds include shallow depth, small surface area, and slow circulation. The water temperature in fish ponds is largely influenced by local air temperature and solar radiation. Due to the periodic fluctuations in air temperature and solar radiation, the water temperature in fish ponds exhibits diurnal and annual variations. This paper studies the diurnal cyclic variations of fish pond water temperature in the summer.

Since both the longitudinal and lateral scales of the fish pond water body in this study are relatively small, a one-dimensional vertical mathematical model was selected for the water temperature field. Assuming the fish pond water is a homogeneous medium with isotropic properties, its thermal characteristics do not change with depth, and there is no lateral heat exchange along the horizontal direction. This allows for simplifying the calculation of the fish pond water temperature field to a temperature field with periodic boundary conditions. The non-steady-state heat conduction equation for this scenario is as follows:

$$\frac{\partial \theta (x,\tau )}{\partial \tau }=a\frac{\partial^{2}\theta (x,\tau )}{\partial x^{2}}$$ (1)

where *τ* is time, *x* is the depth below the water surface, *θ*(*x*,*τ*) is the excess temperature of the water at time *τ* and at depth *x*, *a* is the thermal diffusivity of the water. Thermal diffusivity refers to a physical quantity that indicates the rate of water temperature change under specific heat gain or loss conditions and can be calculated by the following formula.

$$a=\frac{\gamma }{\rho C}$$ (2)

where *γ* is the thermal conductivity of water, *ρ* is the density of water, *C* is the specific heat capacity of water.

Periodic variation boundary conditions have two main characteristics: Due to the periodic variation of boundary conditions, the temperature of various parts of the object is also in a periodic state, so there is no initial condition; The boundary conditions can be regarded as a harmonic wave. According to the fundamental principles of heat transfer theory [28], the excess temperature on the surface of a semi-infinite object can be expressed in the form of a cosine function:

$$\theta (0,\tau )=A_{m}\;\cos(\frac{2\pi }{T}\tau )$$ (3)

where *T* is the fluctuation period, which is 24 hours for diurnal variations, *A*_*m*_ is the amplitude of water surface temperature wave.

By applying the method of separation of variables [29], solving Eq (1) and substituting boundary condition Eq (3), a theoretical computational model for the excess temperature of the water body at different depths and times can be obtained:

$$\theta (x,\tau )=A_{m}\;\exp(-x\sqrt{\frac{\pi }{aT}})\cos(\frac{2\pi }{T}\tau -x\sqrt{\frac{\pi }{aT}})$$ (4)

Let the excess temperature be denoted as $\theta (x,\tau )=t(x,\tau )-t_{m}$, and *t*_m_ is the average temperature with periodic variations. The expression for calculating the temperature field in the fish pond’s water body is as follows:

$$t(x,\tau )=t_{m}+A_{m}\;\exp(-x\sqrt{\frac{\pi }{aT}})\cos(\frac{2\pi }{T}\tau -x\sqrt{\frac{\pi }{aT}})$$ (5)

From Eq (5), it can be inferred that the temperature variation at a depth *x* below the water surface follows a similar pattern to the temperature variation at the water surface itself. In both cases, the temperature changes exhibit cosine functions with the same period. But the amplitude of water temperature fluctuations below the water surface decreases rapidly as the depth *x* increases, following an exponential function. When a certain depth is reached, the amplitude of water temperature fluctuations becomes very small. As the depth *x* increases, the moment when the water temperature reaches its peak gradually gets delayed.

## 3 Materials and methods

### 3.1 FBG temperature sensing principle

**Fig 1:**
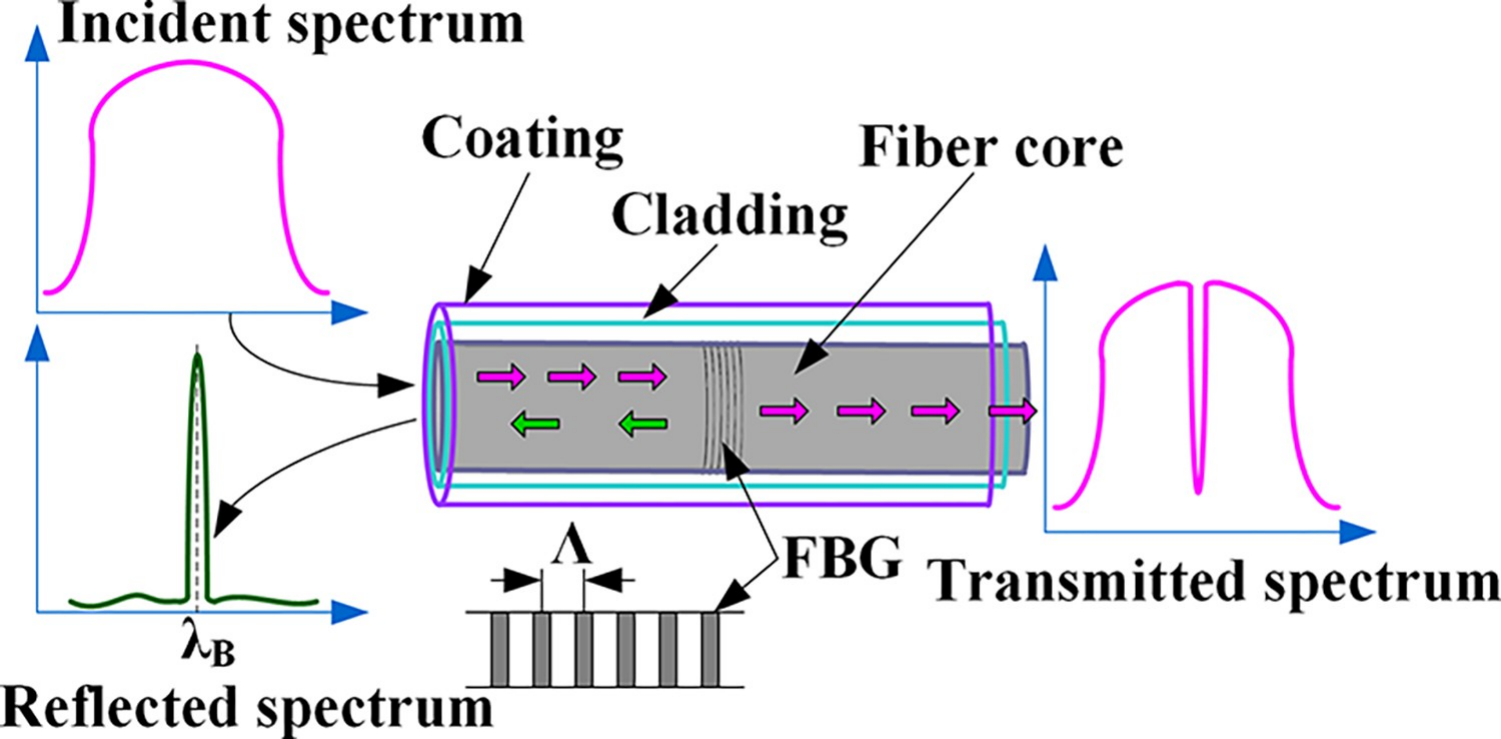
Functional principle of a fiber Bragg grating.

Fiber Bragg Grating is a type of passive filtering device, which is a segment of optical fiber with a periodically varying refractive index in its core. The grating formed by periodic refractive index changes causes diffraction of light, which can reflect specific wavelengths of light, shown in Fig 1. This wavelength is the center wavelength *λ*_B_ of the FBG, which is determined by the effective refractive index *n*_eff_ of the fiber core and the grating period Λ, shown in Eq (6).


$$\lambda_{B}=2n_{\mathrm{eff}}\Lambda$$ (6)

Changes in strain and temperature can lead to variations in the grating period and effective refractive index of the fiber grating, resulting in shifts in the reflected wavelength of the grating. By detecting the shifts in the reflected wavelength of the grating, changes in physical quantities such as strain and temperature can be obtained. In this study, during the arrangement and encapsulation of the fiber Bragg gratings, measures were taken to isolate them from the effects of strain. This way, any changes in the reflected wavelength of the grating solely reflected variations in temperature.

When the temperature of the fiber Bragg grating changes, thermal expansion and thermal-optic effects cause variations in both the grating period and refractive index. The relative displacement of the central wavelength caused by temperature changes Δ*T* is:

$$\frac{\Delta \lambda_{B}}{\lambda_{B}}=\frac{1}{\Lambda }\frac{\partial \Lambda }{\partial T}\Delta T+\frac{1}{n_{\mathrm{eff}}}\frac{\partial n_{\mathrm{eff}}}{\partial T}\Delta T=(\alpha +\xi )\Delta T=K_{T}\Delta T$$ (7)

where $\alpha =\frac{1}{\Lambda }{\frac{\partial \Lambda }{\partial T}}_{T}$ is the thermal expansion coefficient of optical fiber, $\xi =\frac{1}{n_{\mathrm{eff}}}\frac{\partial n_{\mathrm{eff}}}{\partial T}$ is the thermal optical coefficient of optical fiber, *K*_*T*_ is the relative wavelength temperature sensitivity coefficient of the fiber Bragg grating. As indicated by the above equation, it can be inferred that there is a linear relationship between Δ*λ*_B_ and Δ*T*. By detecting the wavelength shift, the temperature change being measured can be calculated.

### 3.2 Water temperature field measurement system based on FBG string

**Fig 2:**
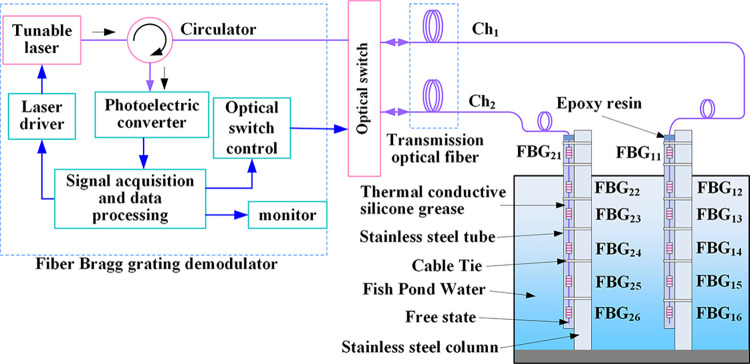
Structural diagram of water temperature field measurement system based on FBG string.

The water temperature measurement system based on an FBG string was shown in Fig 2. Six fiber Bragg gratings with different center wavelengths were inscribed on a single optical fiber using wavelength-division multiplexing technology. FBG_n1_ was employed for measuring ambient air temperature, while FBG_n2_ to FBG_n6_ were utilized for measuring water temperature at different depths underwater. In order to secure the FBGs and ensure precise positioning for measurements, the FBG_n2_ to FBG_n6_ sensors were coated with thermal conductive silicone grease and inserted into stainless steel slender tube. The thermal conductive silicone grease effectively filled the gap between the optical fiber and the stainless steel tube, ensuring consistent temperature between the FBGs and the stainless steel tubes. Fiber Bragg grating sensors encapsulated with stainless steel tubes and thermal conductive silicone grease have a good response speed. Studies have shown that fiber Bragg grating sensors encapsulated using this method can achieve a response speed of 47.1 ms [30]

The upper nozzle of the stainless steel slender tube was sealed with epoxy resin, allowing for the fixation of the optical fiber. The tail end of the FBG string was kept in a freely relaxed state to prevent any stress interference on the FBGs during temperature measurements. During measurements, the stainless steel tubes were affixed to hollow steel columns, which were vertically inserted into the water of the measured fish pond.

Two strings of FBGs were required for the experiment. Switching between the signal channels of two FBG sensor strings was achieved through the utilization of wavelength division multiplexing technology and programmable optical switches. The computer controlled a tunable laser through a driver to generate narrowband laser output. The laser’s scanning frequency and step size were adjustable. The narrowband laser was directed through a circulator and enters the FBG string selected by the optical switch. When the wavelength of the narrowband laser matched the center wavelength of a specific FBG, the reflected light intensity from that FBG became maximum. The reflected light signal reached the photodetector through the circulator, where the optical signal was converted into an electrical signal. The computer utilized a peak detection scanning algorithm to capture the peak values of electrical signals, thereby achieving the localization of FBG measurement points. By calculating the wavelength shift of the reflected light from the FBG, the temperature value of the measurement point could be determined.

### 3.3 Experimental site and experimental design

**Fig 3:**
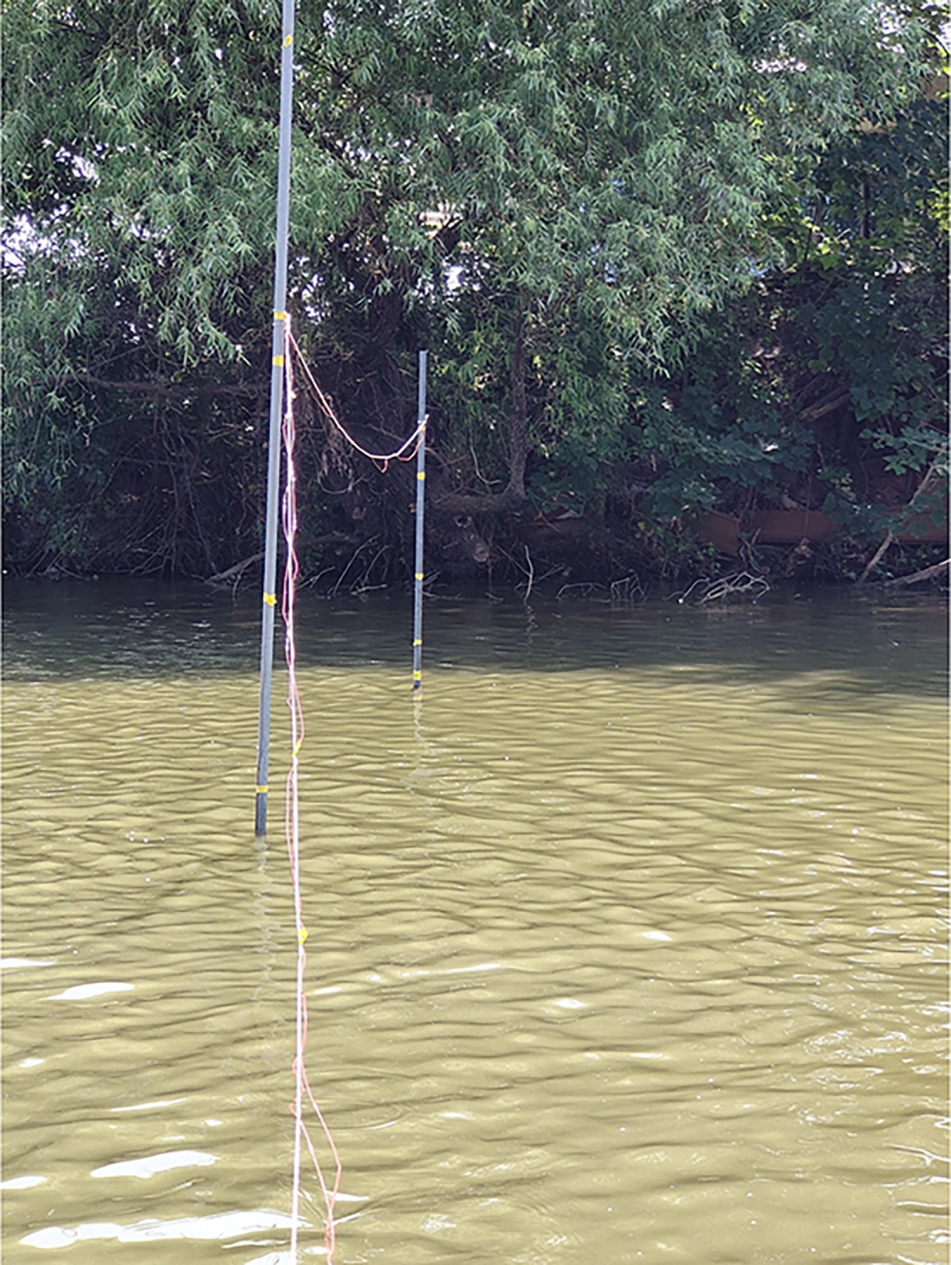
Experimental fish pond.

The experimental site was Pangjia Village Fish Pond in Nanjing City. The experimental fish pond was shown in Fig 3. Its geographical coordinates are latitude 31°49’49"N and longitude 118°57’4"E. The experimental fish pond is an earthen pond with a total area of approximately 2.6 hm^2^ and a water depth ranging from 1.5 to 2.5 meters. The fish species cultured include silver carp, bighead carp, and grass carp, with a stocking density of approximately 1.5 fish/m^2^. The salinity of the pond water is about 0.4‰.The measurements were conducted in August 2023.

The experimental setup was illustrated in Fig 2. The depth range of 50 cm to 100 cm underwater is the primary activity zone for the growth, development, and spawning of freshwater fish in typical aquaculture ponds. In this experiment, FBG_n1_ was exposed to the air at the measurement point for measuring air temperature, while FBG_n2_ to FBG_n6_ were respectively used to measure water temperatures at depths of 5 cm, 20 cm, 50 cm, 80 cm, and 110 cm below the water surface. A solar power meter was placed at the measurement point to simultaneously measure solar irradiance.

The experimental equipment mainly included a FBG demodulator, two FBG strings, and a solar power meter, among others. The central wavelength range of the FBG string is 1530 nm to 1560 nm. The FBG demodulator has a scanning frequency of 100 Hz, covering a wavelength range of 1525 nm to 1565 nm, with a wavelength resolution of 1 pm. Using this demodulator, the author employed an FBG array to measure the surface temperature of photovoltaic modules. The measurement results were generally consistent with those obtained from contact electrical sensors and infrared thermal imagers [27].

In this study, two main types of experiments were conducted: measurements of the daily variation in air temperature and water temperature field, and the investigation of the effects of solar radiation intensity on these temperature fields. In the first type of experiment, the FBG string was positioned 9 meters away from the pond’s shore. The experimental period is from 00:00 on August 10th to 00:00 on August 12th. Data were collected at 5-minute intervals. During the measurement period, the gusty wind speed ranged from 3.1 m/s to 6.7 m/s. Wind speed had a certain impact on air temperature, but its influence on water temperature was minimal.

In the second type of experiment, two strings of FBG sensors were placed at distances of 9 meters and 5 meters from the pond’s shore, respectively. FBG string 1 was utilized to measure the temperature in area A, where there were no obstructions. FBG string 2 was used to measure the temperature in area B, where the sunlight was partially blocked by the tree canopy during the morning hours. The solar power meter was placed at the testing point to measure solar radiation power concurrently. The data collection period was from 6:00 AM to 12:00 PM on August 21st. The weather during the experimental period was predominantly sunny, occasionally partly cloudy.

### 3.4 FBG calibration

**Fig 4:**
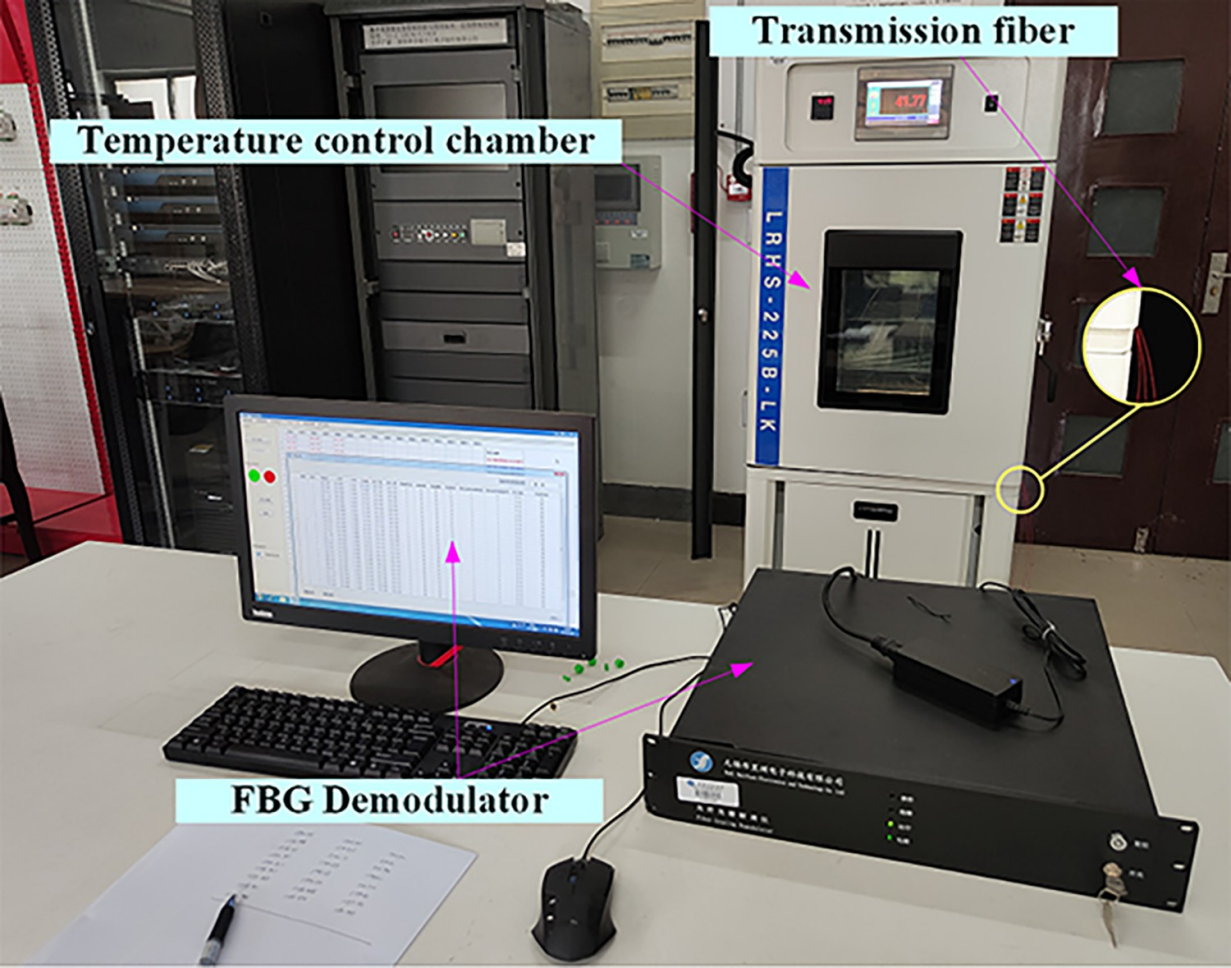
FBG string calibration experiment.

**Fig 5:**
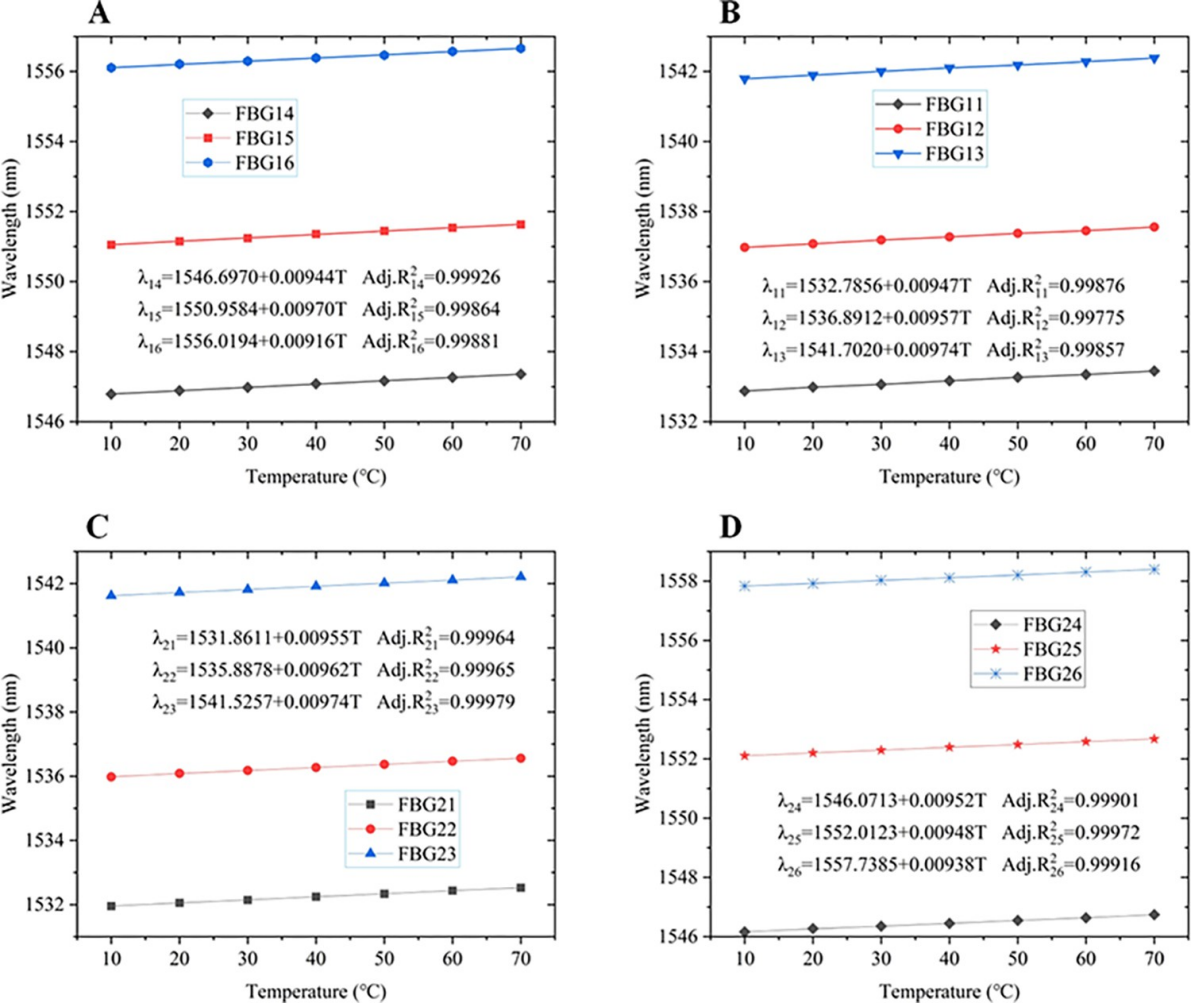
Calibration curves of FBG strings. (**A**) FBG_11_-FBG_13_; (**B**) FBG_14_-FBG_16_; (**C**) FBG_21_-FBG_23_; (**D**) FBG_24_-FBG_26_.

In the experiment, each FBG string contains 6 gratings, totaling 12 FBGs. The grating length for each FBG is 6mm, with a bandwidth of no less than 2nm and a reflectivity greater than 92%. The length of the transmitting optical fiber is 20 m. Prior to the experiment, temperature recalibration was performed on each FBG, as depicted in Fig 4. The FBG string to be calibrated, which is encapsulated with stainless steel tubes and thermal conductive silicone grease, was placed inside a temperature-controlled chamber. The temperature of the chamber was varied within the range of 0°C to 80°C. The central wavelengths of each FBG were recorded at temperatures of 10°C, 20°C, 30°C, 40°C, 50°C, 60°C, and 70°C, respectively. The calibration results for the sensor strings were presented in Fig 5, illustrating that the central wavelength variations of each FBG exhibited a predominantly linear relationship with temperature.

## 4 Results and discussion

### 4.1 Measurement and analysis of daily variations in air temperature and water temperature fields

**Fig 6:**
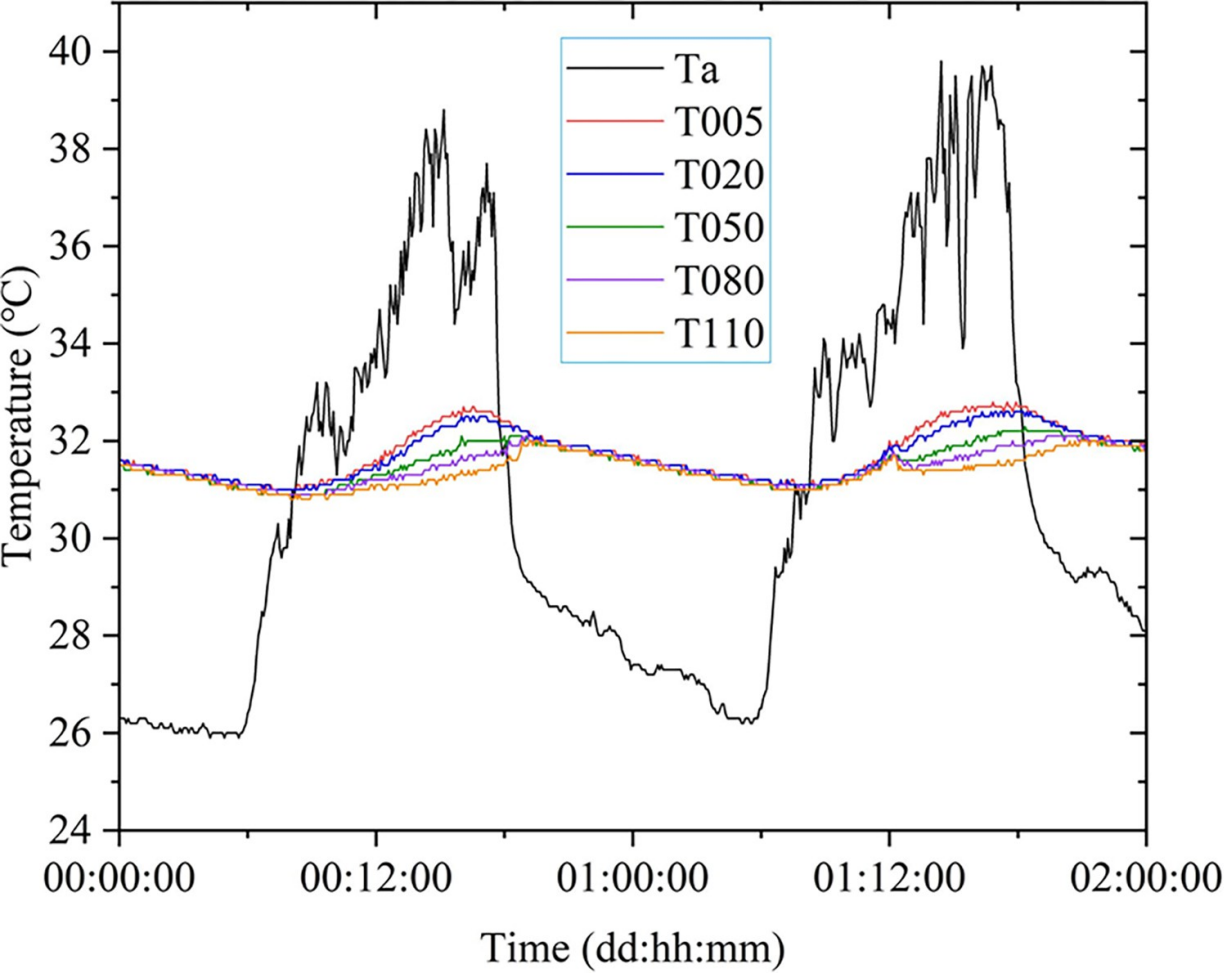
Daily temperature variation curves for air and water in the pond.

The experimental result data can be found in the supporting information, and the daily temperature variation curves of air and water in the pond are shown in Fig 6. T005, T020, T050, T080, and T110 represented water temperatures at depths of 5 cm, 20 cm, 50 cm, 80 cm, and 110 cm, respectively. Ta represented the air temperature at the measurement point. The statistical results of air temperature and water temperature measurements were presented in Table 1.

**Table 1:** Statistical results of daily temperature measurements for air and water.

Measurement Variables	Maximum Temperature (°)	Minimum Temperature (°)	Average Temperature (°)
Ta	39.80	25.90	30.92
T005	32.80	31.00	31.77
T020	32.60	31.00	31.71
T050	32.30	30.90	31.53
T080	32.10	30.90	31.47
T110	32.00	30.80	31.37

By analyzing Fig 6 and Table 1 and referring to the experimental data list provided in the supporting information, the following conclusions can be drawn:

1. The daily variations of air temperature and water temperature followed an approximate cosine function model with a period of 24 hours. Because the specific heat capacity of water was significantly higher than that of air, the amplitude of water temperature changes was smaller than that of air temperature.
2. Influenced by changes in solar radiation and air currents, the daytime air temperature variation curve exhibited more ripples, while the nighttime temperature curve showed fewer ripples. The water temperature’s daily variation curve appeared smoother.
3. The daily average water temperature was no more than 1°C higher than the average air temperature. The average temperature of deeper water was lower than that of shallower water. Between approximately 08:10 AM and 17:50, the air temperature was higher than the water temperature. From around 18:10 to the next day’s 07:50 AM, the air temperature was lower than the water temperature.
4. The highest air temperature during the day occurred between 14:30 and 15:30, while the lowest air temperature occurred between 5:00 AM and 6:00 AM. The times for the daily highest and lowest water temperatures were both delayed compared to the air temperature, and this time lag increased with depth.
5. As the depth increases, the influence of air temperature on water temperature gradually diminished, leading to a decrease in oscillation amplitude. During the daytime, the temperature gradually decreases from the surface to the deeper water layers, whereas at night, the temperature variation among the water layers is minimal. The difference between the maximum water temperature and the minimum water temperature is approximately 2°C.

### 4.2 Impact of solar radiation on air temperature and water temperature field

**Fig 7:**
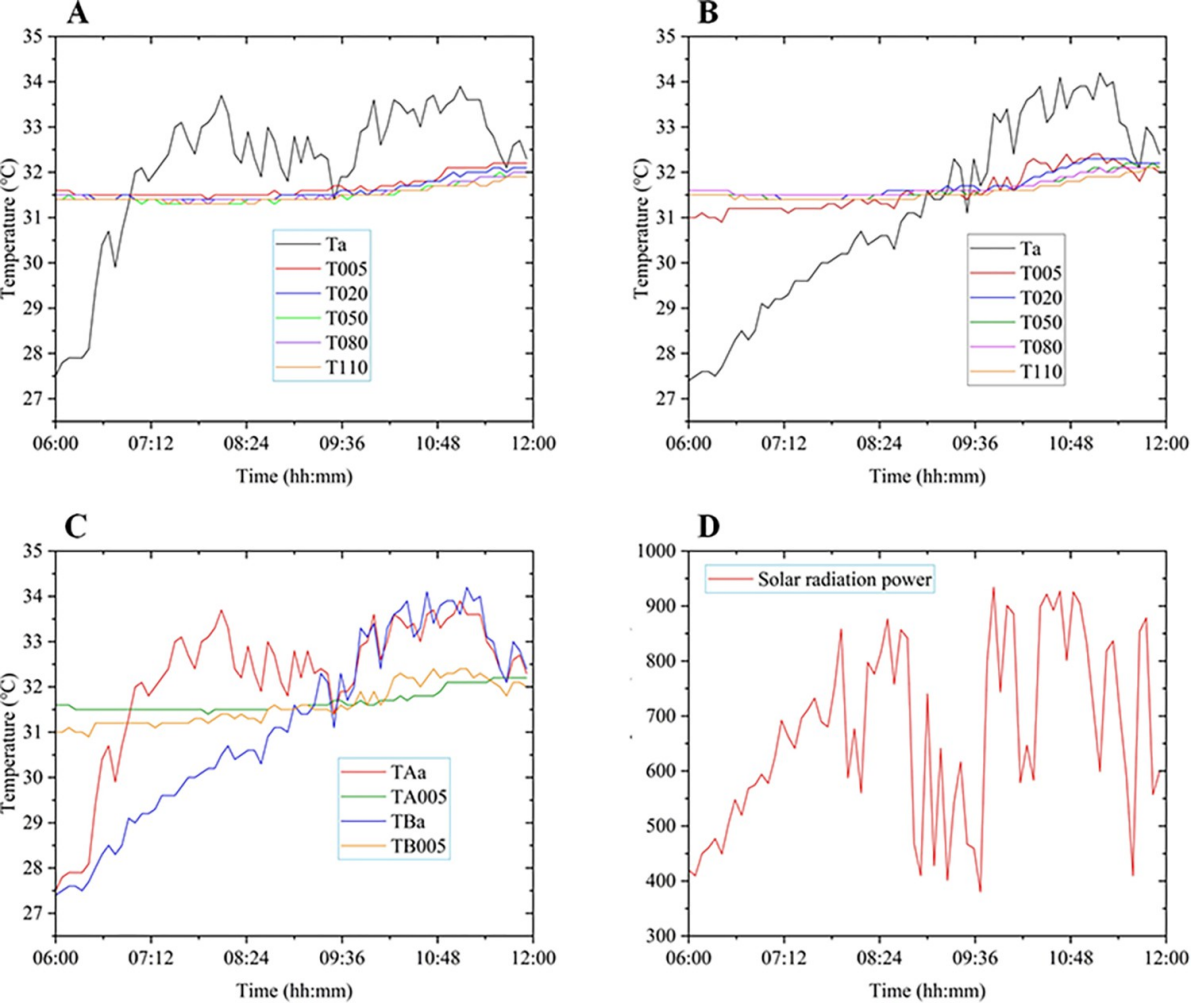
Impact of solar radiation power on temperature field. (**A**) Measurement results of air Temperature and water temperature in zone A; (**B**) Measurement results of air temperature and water temperature in zone B; (**C**) Measurement results of air temperature and surface water temperature in zone A and zone B; (**D**) Measurement results of solar radiation power.

The measurement results were shown in Fig 7.

Analyzing Fig 7(A)–7(C), it could be inferred that: During the period from 6:00 AM to 9:30 AM, area B was shaded by tree canopies, resulting in noticeably lower air temperatures compared to area A. Solar radiation power has a significant impact on air temperature, a certain effect on surface water temperature, but its influence on deep water temperature was not prominent. During this period, the surface water temperature in area A was slightly higher than the deep water temperature due to the influence of solar radiation, while the surface water temperature in area B was significantly lower than the deep water temperature. After 9:30 AM, the sun shone in both areas A and B. The air temperatures in both areas converged, as did the water temperatures. During the period from 7:00 AM to 9:30 AM, affected by solar radiation, the air temperature in area A was significantly higher than the water temperatures, while the air temperature in area B was significantly lower than the water temperatures.

Analyzing Fig 7(C) and 7(D), it could be concluded that: Influenced by fluctuations in solar radiation power, air temperature exhibited more noticeable variations. There was a responsive relationship between temperature and changes in solar radiation intensity.

### 4.3 Analysis of temperature relationships in different water layers

#### 4.3.1 Analysis of the decreasing pattern of temperature amplitude in different water layers

According to the aforementioned theory of temperature distribution, the temperature variations in different water layers of the fish pond exhibited similarities. However, as the depth increases, the amplitude of fluctuations decayed following an exponential function.

**Fig 8:**
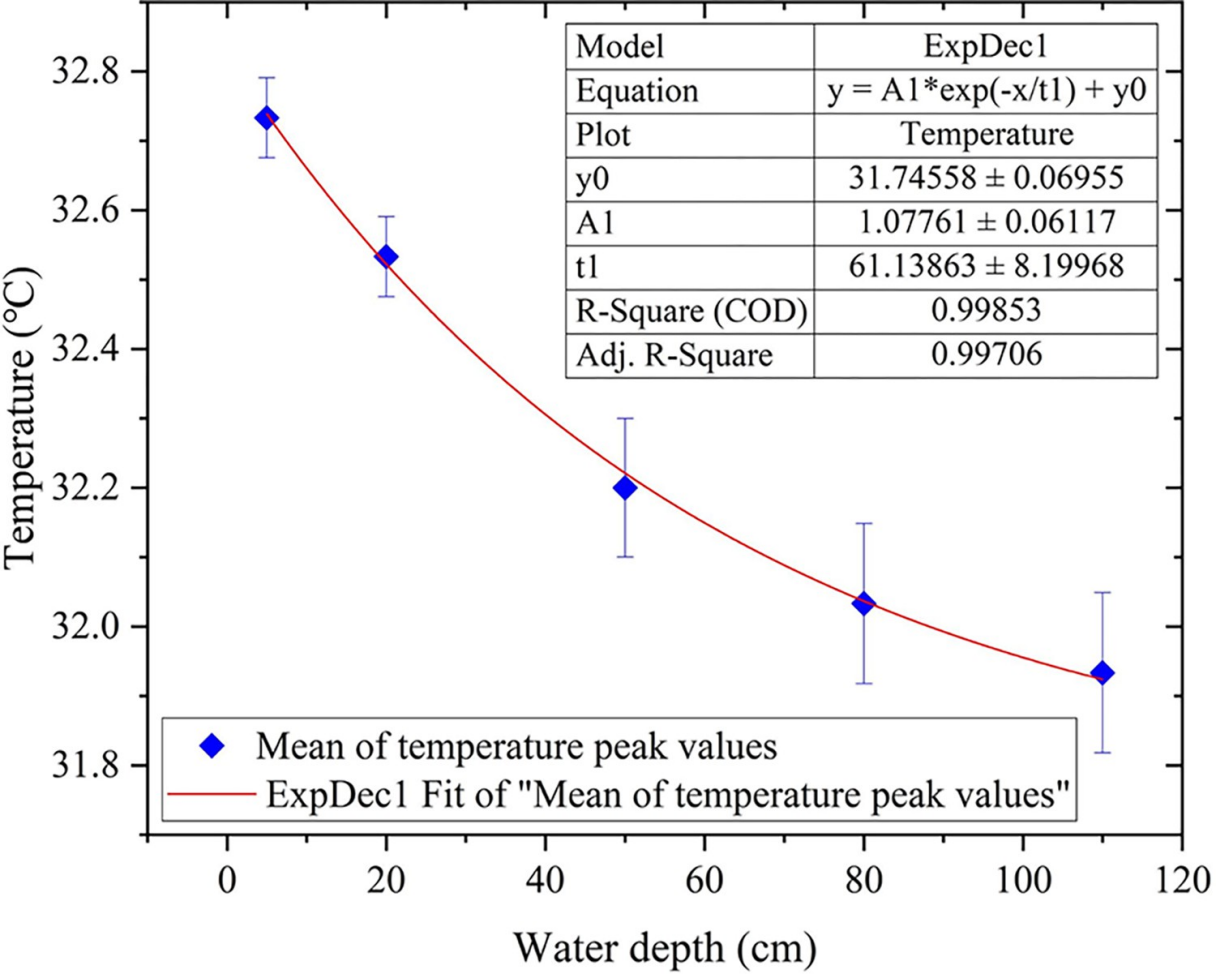
Decay curves of peak temperatures for each water layer.

Observing the temperature variation curves of the pond reveals that the minimum temperatures in each water layer were very similar, while the maximum temperatures occurred between 16:00 and 22:00. The peak temperatures at each depth were collected over three consecutive days in August, and their averages were calculated. A scatter plot was then created to depict the average peak temperature values for each water layer, as shown in Fig 8. To study the decay law of the temperature peak values, the first-order decay exponential function was used to fit the decay trend of the temperature. According to the fitting results, the adjusted fitting degree R^2^ was good, being greater than 0.99.

#### 4.3.2 Correlation analysis of temperatures at different water layers

The Pearson correlation coefficient can measure the direction and degree of the relationship between two continuous variables. Its values range from -1 to 1, where 1 indicates a perfect positive correlation, 0 indicates no linear correlation, and -1 indicates a perfect negative correlation. The Pearson correlation coefficient calculation formula for variables X and Y is as follows:

$$r=\frac{\sum_{i=1}^{n}\left(x_{i}-\bar{x})(y_{i}-\bar{y}\right)}{\sqrt{\sum_{i=1}^{n}{\left(x_{i}-\bar{x}\right)}^{2}}\sqrt{\sum_{i=1}^{n}{\left(y_{i}-\bar{y}\right)}^{2}}}$$ (8)

Where *n* is the number of samples, *x*_*i*_ and *y*_*i*_ are the observed values of variables *X* and *Y* corresponding to point *i*, respectively, $\bar{x}$ and $\bar{y}$ are the sample means of variables *X* and *Y*, respectively.

**Fig 9:**
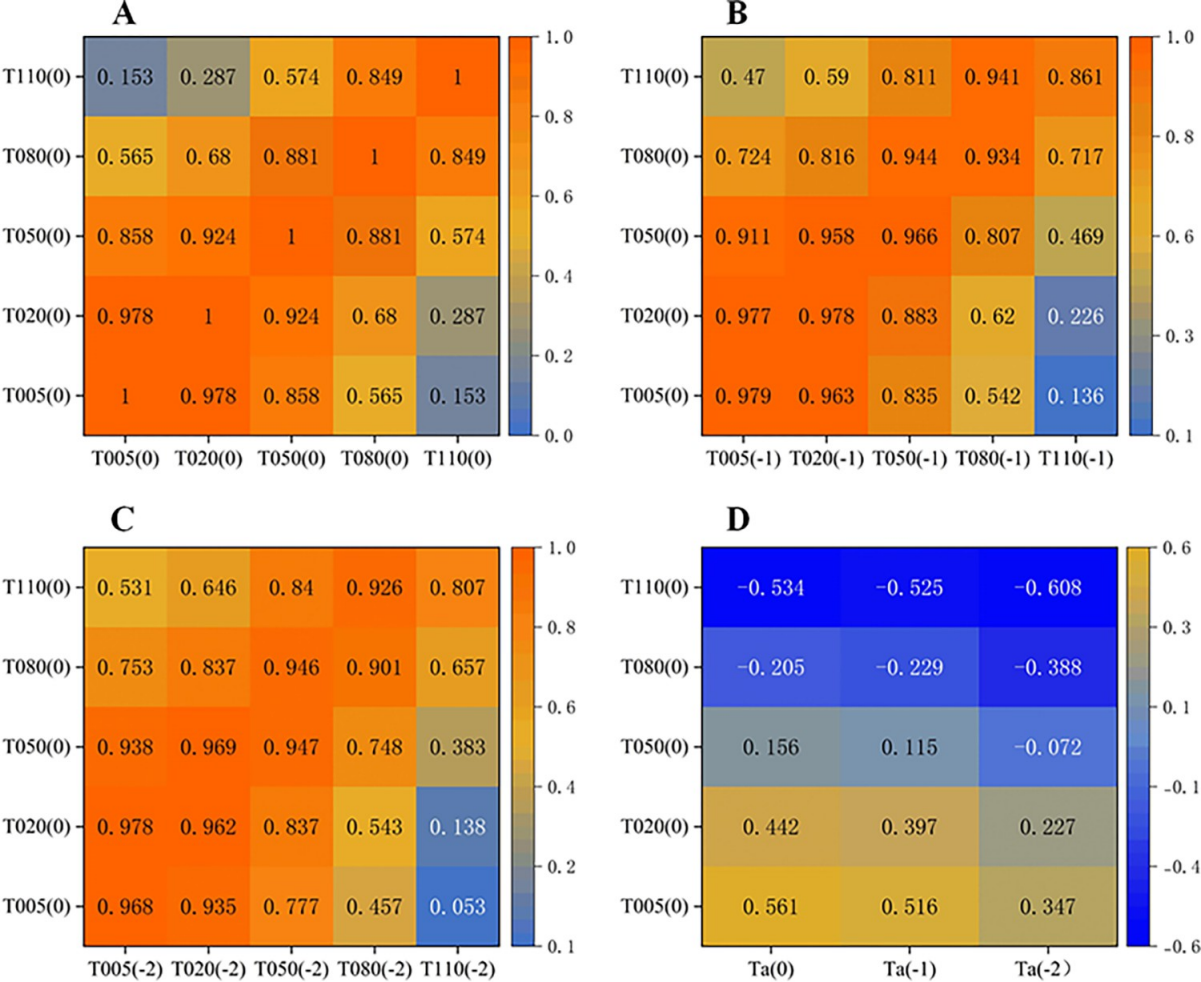
Water temperature correlation heatmap. (**A**) Heatmap of the correlation among water temperatures at different depths for the current day; (**B**) Heatmap of the correlation between water temperatures of the current day and the water temperatures from the previous day; (**C**) Heatmap of the correlation between water temperatures of the current day and the water temperatures from two days prior; (**D**) Heatmap of the correlation between water temperatures of the current day and the air temperatures from the previous 0 to 2 days.

Due to differences in thermal properties such as specific heat capacity, the rate of change of water temperature in relation to air temperature was relatively slow. On a certain day, water temperature was not only correlated with air temperature but also with its previous values. Additionally, there existed correlation among temperatures in different water layers. In this experiment, typical sunny day temperature data for August was selected to study the linear correlations among water temperatures at various depths below the water surface, as well as the linear correlations between these water temperatures and air temperature. The linear correlation degree was represented by the Pearson coefficient, and the statistical results are shown in Fig 9. In the figure, "(0)" represents the current day, "(-1)" represents the previous day, and "(-2)" represents two days prior.

Analyzing Fig 9(A), it could be observed that there was a correlation among water temperatures at different depths on the same day. The correlation was stronger when the distance between water layers was smaller. For instance, T05 was highly correlated with T020 and T050. T020 and T050 were also highly correlated, T05 and T080 were moderately correlated, and T05 and T110 were not significantly correlated.

Analyzing Fig 9(B) and 9(C), it could be observed that the water temperature at a certain depth on a given day was not only correlated with the water temperature at the same depth from the previous day, but also with the water temperatures at shallower depths from the previous day and the day before that. For example, T50(0) was correlated not only with T50(-1), but also with T20(-1), T20(-2), T05(-1), and T05(-2). This reflected the characteristic of solar radiation and atmospheric heat radiation being transferred from the water surface to deeper layers during the summer.

Analyzing Fig 9(D), it could be observed that the water temperature at the surface layer was moderately correlated with the air temperature of the current day. However, the correlation between the water temperature at deeper layers and the air temperature was smaller and even negligible. The correlation between the water temperature at the surface layer and the air temperature of the current day, as well as the previous 1 and 2 days, gradually decreased

## 5 Conclusions

In this study, a temperature measurement system based on a fiber Bragg grating string was designed. In this system, six FBGs with different central wavelengths were serially connected on a single optical fiber, which was then enclosed in a stainless steel tube and vertically fixed in the water of a fish pond. The system was used for fish pond water temperature measurement experiments, and the distribution and variation of the water temperature in the fish pond during the summer had been obtained. The experimental results are as follows:

1. During the summer season in the fish pond, the daily variation curve of water temperature in each layer exhibits an approximately cosine function with a period of 24 hours. The daily variation curve of water temperature is relatively smooth. The amplitude and rate of temperature change in water are both lower than that of air temperature. In summer, the daily average water temperature in the pond is no more than 1°C higher than the average air temperature. The average temperature in the deeper water layers is lower than that in the shallower layers. The timing of the highest and lowest water temperatures also lags behind the air temperature.
2. During the daytime, the temperature gradually decreases from the surface to the deeper water layers, whereas at night, the temperature variation among the water layers is minimal. As the depth increases, the oscillation amplitude of the water temperature curve gradually decreases, and the amplitude follows an exponential decay function. However, the peak time of the oscillation gradually lags behind. The difference between the maximum water temperature and the minimum water temperature is approximately 2°C.
3. Solar radiation has a significant impact on air temperature, and there is a corresponding relationship between air temperature and variations in solar radiation intensity. Solar radiation also has a certain influence on surface water temperature, but its impact on deep water temperature is not pronounced.
4. The temperatures of different layers in the fish pond are correlated, and the correlation is stronger when the distance between water layers is smaller. The water temperature at a certain depth on a given day is correlated with the water temperature at the same depth from the previous day. Furthermore, it is also correlated with the water temperatures at shallower depths from both the previous day and the day before that. The temperature of the surface water layer is moderately correlated with the current day’s air temperature, while the deep water temperature has little or no correlation with the air temperature.

In this study, real-time measurement of the vertical water temperature field in a fish pond was achieved using a fiber Bragg grating string measurement system. The experimental results provide data support for scientific research, as well as for aquatic planting and aquaculture. This measurement method also offers reference and guidance for measuring temperature fields in other fluids.

This study obtained the distribution and changing patterns of the water temperature field in a summer fish pond in the suburb of Nanjing. However, to more accurately grasp the spatiotemporal variation of the temperature field in fish ponds, longer-term monitoring is needed.

However, when using the method proposed in this paper to encapsulate the fiber Bragg grating string for long-term underwater temperature monitoring, the issue of thermal conductive silicone grease degradation should be taken into account. Thermal conductive silicone grease that has exceeded its shelf life undergoes fundamental changes in its thermal and mechanical properties, requiring timely replacement, and the fiber Bragg grating string also needs to be re-encapsulated.

## Supporting information

S1 Data Figs 4–8 supporting data. (XLSX)
